# Core and occasional species: A new way forward

**DOI:** 10.1002/ece3.7863

**Published:** 2021-07-07

**Authors:** Alvin J. Helden

**Affiliations:** ^1^ Applied Ecology Research Group School of Life Sciences, Anglia Ruskin University Cambridge UK; ^2^ UCD School of Agriculture and Food Science University College Dublin Dublin 4 Ireland

**Keywords:** community structure, core and occasional species, grassland insects, multimodality, Simpson's index

## Abstract

1. Various methods have been used to divide communities into core species and occasional or satellite species. Some methods are somewhat arbitrary, and there is evidence that many communities are more multimodal than bimodal. They also tend to rely on having multiple years of data.
2. A completely novel method is presented that not only has no requirement for long‐term datasets but can divide communities into multiple groups. It is based on probability a species is present, calculated using Simpson's index and the sequential removal of species from the data.
3. The sequential Simpson's index method was applied to species data from a grassland insect community. It was also applied to eleven other datasets that had been divided into core and occasional species in previously published studies.
4. The new method was found not only to be consistent with previous core–occasional assessments but also able to identify multimodality in species abundance distributions.
5. Although ideally used with a measure of persistence (frequency of occurrence) to rank species, community structure is consistently described even with only species abundance data.
6. The method can be applied to short or long‐term datasets and can help identify multimodality and provide valuable insight into how communities change in time or space.

## Graphical abstract

This paper describes a novel method of dividing ecological communities into species groups based on their relative abundance. It builds on the core–occasional species concept but tackles grouping of species in a completely new, more objective way, with no a priori assumption of the number of groups in a community. It shows strong agreement with previous studies but is not reliant on long data series, so can be applied with a more fine‐grained approach to study temporal and spatial variation.

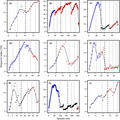

## 1 INTRODUCTION

The idea of dividing up communities into species groups, based on their relative abundance or frequency of occurrence (persistence), is long standing (e.g., see Winterbottom, 1949). However, it was arguably not approached in a more formalized way until the core‐satellite hypothesis of Hanski (Hanski, 1982; Magurran, 2007; Supp et al., 2015), in which distribution the core species are found at more sites and are relatively abundant, compared with satellite species. The division of species into groups based on the frequency that species are encountered was incorporated into the UK's National Vegetation Classification (NVC), in which plant species were classified into five frequency classes, based on 20% bands (Rodwell, 2006). In the NVC, the two highest frequency bands were given the term, constant, with the other three groupings being, frequent, occasional, and scarce.

Magurran and Henderson (2003) extended the concept by replacing spatial distribution with temporal frequency and not applying any a priori expectation of frequency classification. They used data from a 21‐year study of fish in the Bristol Channel, UK. By plotting maximum yearly abundance against number of years, a species was recorded (persistence); they were able divide the community into core and occasional species. Core species were more persistent and generally more abundant and ecologically associated with estuarine habitats. Occasional species were infrequent, typically less abundant and not considered estuarine species.

The deconstruction of communities into core and occasional groups is valuable as it may enable changes in the abundance of individual species or species groups to be detected (Magurran & Henderson, 2010; Whittaker, 2015). It can also be used to assist in the study of species abundance distributions (SADs) and the testing species abundance models (Magurran, 2007). Although a number of models have been proposed to describe SADs (Antão et al., 2017; Magurran, 2004), it has become clear that SADs are frequently not adequately described by them, at least in part due to their multimodal nature (Antão et al., 2017; Matthews, Borges, et al., 2014).

Multimodality can be defined as the presence of two or more species groupings within communities (Antão et al., 2017; Matthews, Borges, et al., 2014; Ugland & Gray, 1982). One way of understanding multimodality is to develop alternative models to describe SADs, such as the Gambin model (Matthews, Borregaard, et al., 2014; Ugland et al., 2007; Whittaker, 2015). Another approach is to identify the groupings that generate multiple modes and study their characteristics.

Since the early work of Hanski (1982), and Magurran and Henderson (2003), many studies, using a range of approaches, have deconstructed communities into occasional or satellite, and core species. The communities studied have been varied, covering a wide range of taxa (Astudillo‐García et al., 2017; Coyle et al., 2013; van der Gast et al., 2011; Genner et al., 2004; Gray et al., 2005; Ulrich & Ollik, 2004; Ulrich & Zalewski, 2006; Umaña et al., 2017).

Among these studies, Genner et al. (2004) used a modification of Magurran and Henderson's (2003) method to identify core and occasional species of fish in long‐term data from the Bristol and English Channels. Plotting abundance against persistence, they modeled the relationship with a third‐order polynomial and divided the community at the inflection point into core and occasional species, with the same Bristol Channel community sub‐division as Magurran and Henderson (2003). Ulrich and Ollik (2004) classified core species of forest Hymenoptera as being those found in six or more years from eight, while occasional species were found in three or less years. Several other studies have taken a similar, rather arbitrary approach by defining core species at being one end of a measure of frequency and occasional the other end (Coyle et al., 2013; Hansen & Carey, 2015; Ulrich & Zalewski, 2006). Some have used a single frequency point to divide communities into two groups (Barnes et al., 2016; Dolan et al., 2009; Matthews, Borges, et al., 2014), while others have considered the presence of intermediate species (Boss & Silva, 2014; Supp et al., 2015). Alternative methods have included using species' variance to mean ratio, calculating Simpson's index for subgroups of species and fitting mathematical models to community data (Boisnier et al., 2010; van der Gast et al., 2011; Gray et al., 2005). There has been criticism that many approaches have been arbitrary and a potential source of artifacts, although some studies have tried to use biological characteristics of the communities to help improve the reliability of species groupings (Barlow et al., 2010; Boisnier et al., 2010; Whittaker, 2015). As well as arbitrariness, any attempt to divide a community into core and occasional species presupposes the hypothesis that a binary division best describes a community. However, such a simple a priori approach may not reflect the reality and complexity of many, or indeed most communities, and their arbitrary nature makes replication of methods problematic. Indeed, with multimodality believed to be widespread (Antão et al., 2017; Dornelas & Connolly, 2008), applying a bimodal description to a community risks missing potentially interesting ecological patterns. Therefore, there is a need for an approach to divide communities in a more descriptive, objective, and reproducible way.

Another limitation of some previous methods has been that core–occasional and core‐satellite distinctions have relied on having very large datasets, only available after sampling for many years or at many sites, thus precluding their use from shorter term or smaller scale studies. For example, Magurran and Henderson (2003) was based on 21 years of sampling fish communities. Deconstructing communities without such runs of data is more difficult, and unfortunately, much community data from field research are short‐term (Barlow et al., 2010). Finding a reliable method for objectively identifying species groupings without long‐term/multiple site sampling would therefore be very valuable. It would allow many more community datasets to be studied in terms of species groupings and would have the additional benefit of making it easier to look at how SADs and changes to species groupings vary over time or space (Magurran, 2007; Magurran & Henderson, 2010).

I studied the grassland insect community from an intensively cattle‐grazed site at Teagasc Grange, Ireland, over 4 years, to which I attempted to apply a binary core–occasional partitioning of species. After an initial use of a modified form of the Genner et al. (2004) approach, in which I identified the method of calculating persistence as being potentially problematic, I developed a novel approach based on calculation of the Simpson's index and the sequential removal of species from the data. The new method has the advantage over previous partitioning methods of being descriptive and making no a priori assumption regarding the pattern of species groupings within a community, thus moving away from a simple binary division on communities. It can also be applied to a much wider range of datasets, as it is much less reliant on having data from multiple years or sites. The method was tested against data from Magurran and Henderson (2003) and Genner et al. (2004), as well as nine other datasets from previous studies. In this paper, I describe this new method and discuss its application to the study of community structure. Specifically, I test the following questions:

1. Is it sensitive to the method of calculating persistence (frequency of occurrence)?
2. Does it show the same pattern a species grouping when species are ranked by persistence or by abundance?
3. Can it be applied to a range of previous community datasets?

## 2 MATERIAL AND METHODS

### 2.1 Insect sampling

Coleoptera and Hemiptera were suction sampled (*n* = 692) from the vegetation and ground surface of a cattle‐grazed agricultural grassland (Drennan & McGee, 2009; Helden et al., 2015) at Teagasc Grange, Co Meath, Ireland, between 2002 and 2005. Further details of sampling and species identification can be found in Helden et al. (2015).

### 2.2 Statistical modeling and index calculations

The statistical modeling and calculation of values for Simpson's index, described below, were carried out using R version 3.4.0 (R Core Team, 2017). Code used for data manipulation and subsequent calculation of Simpson's index is given in Appendix A.1.

### 2.3 Application of Genner et al. method to Grange Hemiptera and Coleoptera data

The Genner et al. (2004) method was applied to Grange Hemiptera and Coleoptera species and abundance data, to divide the species into core and occasional species (i.e., a binary classification). With only 4 years of data, year could not be used as a useful measure of persistence. Instead, it was measured by the number of times a species was found in a group of samples. To take account of phenological change, samples from all years and dates were randomly allocated in approximately equal‐sized groups. Group size was defined by the number of individuals rather than number of samples. The minimum group size was taken as the number of individuals required to give a stabilized value (i.e., groups of the same or larger size would show no difference) of Simpson's index (1/*D*) (Lande et al., 2000; Magurran, 2004). For Hemiptera, this was a mean group size of 43.6 individuals (115 groups) and for Coleoptera 99.7 individuals (63 groups). For each species, persistence was determined as the number of groups in which it was present.

Log_10_ mean abundance for each species was plotted as the response and persistence as the explanatory variable to give a sigmoidal pattern. A third‐order polynomial line (*y* = *x* + *x* ^2^ + *x* ^3^) was fitted, and the value for persistence at the inflection point was determined. Species were classified as core if more frequent than the persistence value observed at the inflection point and as occasional if less so.

Although minimum group size could be determined using Simpson's index (see above), it was not known what the effect of larger group sizes would be on the number of core species. Therefore, the procedure was repeated for a series of nine other group sizes for both Coleoptera and Hemiptera. For Coleoptera, these were 146.1 (mean) individuals (63 samples); 202.7 (31); 251.3 (25); 299.2 (21); 349.1 (18); 418.9 (15), 448.8 (14), 523.6 (12), and 628.3 (10). The equivalent for Hemiptera was 94.5 (53); 147.3 (34); 200.4 (25); 250.5 (20); 313.1 (16); 357.8 (14); 417.4 (12); 455.4 (11); 500.9 (10).

### 2.4 Proposed new sequential Simpson's index method applied to Grange insect data and to Bristol and English Channel fish data

The proposed new sequential Simpson's index method was applied to Hemiptera and Coleoptera data from Grange, as well as to the Bristol Channel and English Channel fish data from Genner et al. (2004).

For Grange, the minimum group size (as defined in the previous section, above) was used for Hemiptera (115 groups, mean abundance 43.6) and Coleoptera (63 groups, mean 99.7). The frequency that a species was found in the groups was used only to rank species in terms of persistence. Species of equal persistence were ordered by abundance. Simpson's index (1/*D*) was calculated for the full community after adding one to the abundance of each species. Then, the highest ranked species (most persistent) was removed and Simpson's index recalculated. This was repeated with the sequential removal of the most persistent until only one species remained, when Simpson's 1/*D* = 1.

Simpson's index was plotted as the response variable with species rank (persistence) as the explanatory variable. The resultant data were modeled with polynomial generalized linear models using Gaussian error structure. A series of models, beginning with 1/*D* + (1/*D*)^2^, were created. For each new model, the next sequential power was added, such that the next was a third order polynomial, followed by a fourth order polynomial, and so on. The proportion of deviance explained by each model was calculated using the Dsquared function in the modEvA package (Barbosa et al., 2015). Increasing the polynomial power led to an increase in deviance explained, up to a point at which the deviance showed little further increase. The first model as deviance plateaued was taken as the optimal model. Model simplification was then carried out by the sequential removal of any nonsignificant terms, with adjacent models compared with AIC values and deletion tests used to justify changes (Crawley, 2007).

Polynomial models showed linear alternation in concavity at inflection points. These were identified by solving the model for each species rank and determining between which ranks the model concavity changed (Appendix A.1). The inflection points were taken as indicating dividing points between species groups. Original abundance and persistence data were compared with the inflection points. In some cases, at the less abundant and infrequent end of the data, species groups were combined when abundance and/or persistence were identical or very similar. In some cases when adjacent species near group boundaries had identical persistence, the boundary of the grouping was moved slightly away from the inflection point itself. The multiple species groupings form a continuum from the most persistent at one extreme to the least persistent at the other. The data were also inspected to try to determine a suitable dividing point between core (C) and occasional (O) species groups. This was judged in part by the position of the inflection points and partly by the abundance and persistence of the species. In most datasets, this was either at the central inflection point, in the case of an even number of groupings, or one of the two central inflection points for an odd number.

To test whether the number of core species defined by the new method was sensitive to group size, it was tested using the same 10 groups of Grange Coleoptera and 10 groups of Hemiptera data, as described in the previous section. This enabled the method to be compared with the Genner et al. (2004) method in terms of group size effect.

The sequential Simpson's method was then applied to the same four datasets but instead of ranking species by persistence, they were ranked by abundance alone. This was because in many datasets, based on single sampling events, it is not possible to rank according to persistence. Species near boundaries were compared and if of equal abundance the boundary was adjusted accordingly.

### 2.5 Application of new sequential Simpson's index method applied to nine published datasets

The new method was applied to nine datasets from seven papers. These were as follows.

1. The average abundance and number of sites occupied by *Onthophagus* (Coleoptera: Scarabaeidae) in Sarawak, Malaysia, grouped as core or satellite by Hanski (1982). Data extracted from figures 4 and 9 in Hanski (1982).
2. Tree species abundance from Barro Colorado Island (BCI), Panama, classified as common or rare by Gray et al. (2005). Species groupings were derived from the BCI‐50 plot in figure 3b of Gray et al., 2005, with original species data from Condit et al. (2012).
3. Carabidae (Coleoptera) abundance and number of sites found in Poland. Data from table 1 of Ulrich and Zalewski (2006).
4. The total abundance and number of days sampled for tintinnid ciliates (Ciliophora: Choreotrichia: Tintinnida) in the Mediterranean Sea. Data extracted from figure 3 of Dolan et al. (2009).
5. The persistence and abundance of fish species from an artificial marine reef in California, USA, which were divided into core and transient groups by Boisnier et al. (2010). Original data from table 5 of Matthews (1985).
6. Boisnier et al. (2010) also used a second dataset, from a study of fish from an artificial marine reef in Australia. The data from table 2 in Branden et al. (1994).
7. Two datasets, phytoplankton and fish from a lake in Wisconsin, USA, were divided into core, intermediate, and occasional species by Hansen and Carey (2015). Data from supporting information S1 table in Hansen and Carey (2015).
8. Abundance data of small mammals from Arizona, USA, divided into core, intermediate, and transient by Supp et al. (2015). Data were available from table 1 in Supp et al. (2015).

All were ordered by the frequency of species being found. For data from Hanski (1982), BCI (Gray et al. 2005), and Ulrich & Zalewski (2006) this was in terms of the number of sites a species was present, with the number of times a species was found for the remainder.

## 3 RESULTS

### 3.1 Effect of group size on classification of core species—comparison of methods

**FIGURE 1:**
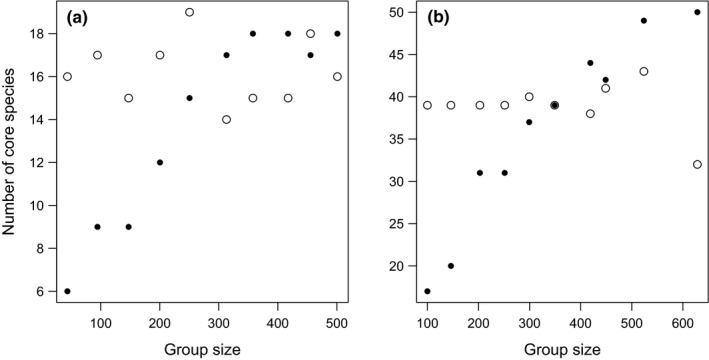
The effect of group size on the number of species classified as core species, using the technique of Genner et al. (2004) (filled circles) and the proposed new sequential Simpson's index method (open circles); with group size being the number of individuals allocated to a group to enable the calculation of persistence (frequency of occurrence). Data come from samples from Teagasc Grange: (a) Hemiptera; (b) Coleoptera

With the Genner et al. (2004) method, the number of core species classified showed a strong positive relationship with the size of the grouping used as a measure of persistence (Spearman rank correlation: Hemiptera *r_s_* = 13.22, *n* = 10, *p* < 0.001; Coleoptera *r_s_* = 2.51, *n* = 10, *p* < 0.001) (Figure 1). For Hemiptera, the smallest group size (mean 43.6 individuals per group) resulted in six species and the largest (500.9) 18 species, classified as core (Figure 1a). For Coleoptera, the equivalent figures were 17 (100.0) and 50 (628.3) (Figure 1b). In contrast, there was no significant effect of group size using the propose new sequential Simpson's index method (Spearman rank correlation: Hemiptera *r_s_* = 177.22, *n* = 10, *p* < 0.839; Coleoptera *r_s_* = 157.53, *n* = 10, *p* = 0.901) (Figure 1).

### 3.2 Proposed new sequential Simpson's index method applied to different community datasets

The following description of the results focuses on the overall patterns, particularly in terms of the core–occasional species split. More detailed description of the model output for each dataset can be found in Appendix A.2.

### 3.3 Bristol Channel fish

**FIGURE 2:**
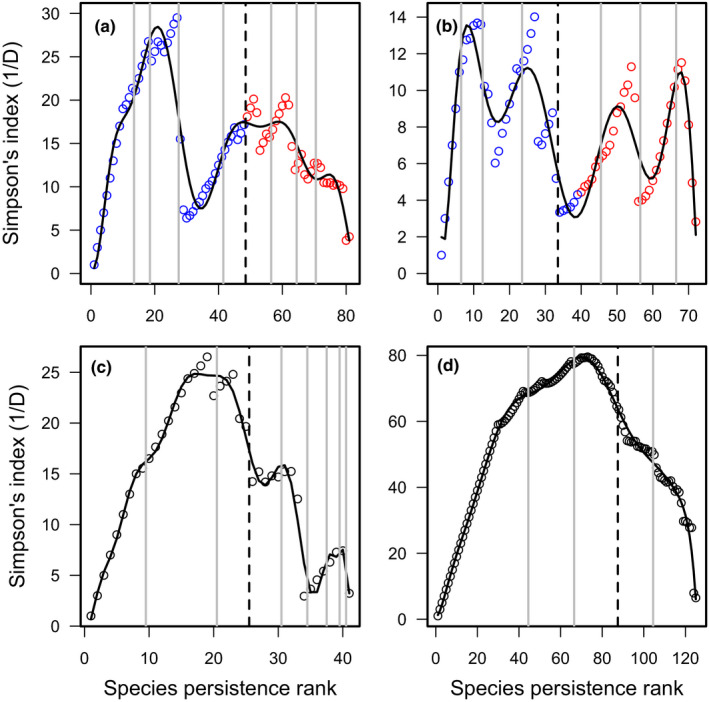
Simpson's index (1/*D*) values calculated from species abundance data ranked by persistence (frequency of occurrence) with the sequential removal of the most persistent species. Solid lines show the best fitting polynomial models. Vertical lines show the boundaries between species groups, with the dashed line indicating the division between core and occasional species groups. Species groupings indicated by Genner et al. (2004) are shown as red for core and blue for occasional. (a) Bristol Channel fish; (b) English Channel fish; (c) Grange Hemiptera; (d) Grange Coleoptera

**FIGURE 3:**
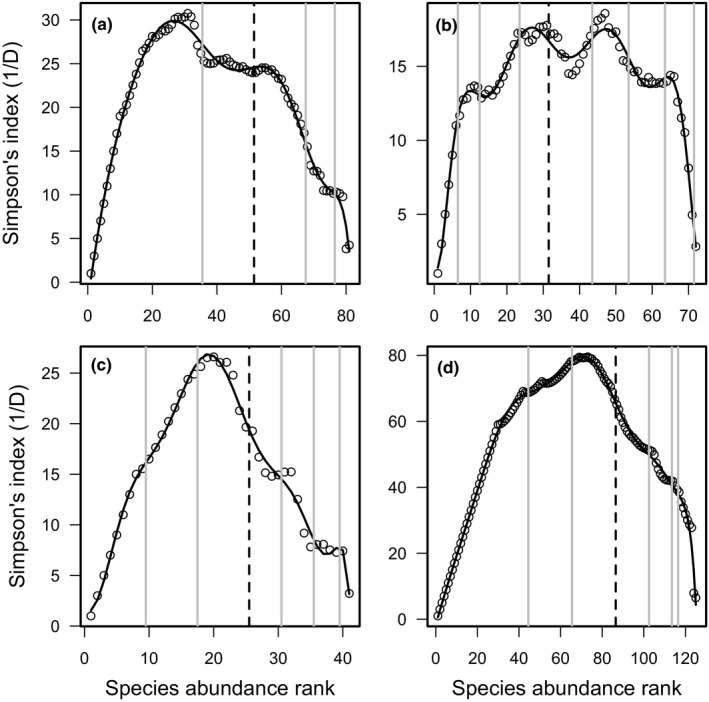
Simpson's index (1/*D*) values calculated from species abundance data ranked by abundance with the sequential removal of the most abundant species. Lines show the best fitting polynomial models. (a) Bristol Channel fish; (b) English Channel fish; (c) Grange Hemiptera; (d) Grange Coleoptera

When ranked by persistence, the community was divided between 33 core species within four groups, and 48 occasional species in five groups (Table 1, Figure 2a), which was identical to the 33–48 split of Genner et al. (2004). When ranked by abundance, 30 species were classified as core (in three groups) and 51 as occasional (two groups). The ratio of core and occasional species, when ordered by persistence and by abundance using the new method, showed no significant difference ($\chi_{1}^{2}$ = 0.234, *p* = 0.629). The abundance‐based division was also not significantly different than that of Genner et al. ($\chi_{1}^{2}$ = 0.234, *p* = 0.629). Although there was little difference between the core–occasional split between persistence ranked and abundance ranked data, the subgroups did show differences in size and number (Figures 2a, 3a).

**TABLE 1:** Species groupings of the two fish and two insect communities deconstructed using the sequential Simpson's index method, after being ranked by persistence

	Species grouping											% of individuals in core groups
	C1	C2	C3	C4	C5	C6	O1	O2	O3	O4	O5	
Bristol Channel fish	11	6	8	8	—	—	7	14	9	5	13	99.4%
English Channel fish	6	10	11	12	—	—	10	11	6	6	—	99.7%
Grange Hemiptera	1	1	2	3	4	5	5	11	9	—	—	98.5%
Grange Coleoptera	21	17	—	—			21	22	44	—		94.7%

Note

C = core species, O = occasional species.

### 3.4 English Channel fish

Modeling of persistence ranked data resulted in 39 core species in four groups and 33 occasional species also in four groups (Table 1, Figure 2b), which was not significantly different from the 33–39 split of Genner et al. ($\chi_{1}^{2}$ = 1.000, *p* = 0.317). The division between core and occasional species with was not significantly different from data ordered by abundance (41 core and 31 occasional species) ($\chi_{1}^{2}$ = 0.113, *p* = 0.737) or from the classification of Genner et al. (2004) ($\chi_{1}^{2}$ = 1.779, *p* = 0.182). The number and size of species groupings were very similar between persistence ranked and abundance ranked data (Figures 2b and 3b).

### 3.5 Grange Hemiptera

The persistence ranked community was split into six core groups containing 16 species and three occasional groups with a total of 25 species (Table 1).

The core group with the greatest persistence contained a single species of grass‐feeding aphid, *Rhopalosiphum* that was clearly more abundant and more frequent than all other species (Appendix A.3). The next most persistent core group also contained a single grass‐feeding aphid, *Metopolophium*, much less abundant but still found in 102 of the 115 groups. Further details of the boundaries of each core and occasional group, in terms of persistence and abundance figures, are shown in Appendix A.3.

One species of *Javesella* (Delphacidae) was in the fourth core group, another in the fifth, and a third was in the occasional group with the highest persistence. Similarly, the genus *Macrosteles* (Cicadellidae) had one species in the fourth core, one species in fifth core, and one in sixth core group. Details of the species within each group can be found in Appendix A.4.

Modeling of abundance ranked data resulted in classification of 15 core and 26 occasional species. There was no significant difference between the number of species categorized as either core or occasional between the persistence ranked and abundance ranked data ($\chi_{1}^{2}$ = 0.203, *p* = 0.652). There was also no difference in the categorization of the subgroup size (Fisher's exact test *p* = 0.889).

### 3.6 Grange Coleoptera

The best fitting model for persistence ranked data divided the community into five species groups: two core groups, with a total of 38 species, and three occasional, with 87 species (Table 1). Details of the boundaries of each core and occasional group, in terms of persistence and abundance figures, are shown in Appendix A.3.

Two species, *Mocyta fungi* and *Amischa analis,* occurred in all possible sample groups and were both far more abundant than any other species. There were 11 species from the genus *Stenus* (Staphylinidae). Four in the most persistent core group, three in the next group, three in the first occasional group, and one in the next (Appendix A.4). There were three Curculionoidea that specialize on feeding on *Trifolium*: *Sitona lepidus* and *Protapion fulvipes* in the most persistent core group and *Ischnopterapion virens* in the most frequent occasional group. Details of species groupings can be found in Appendix A.4.

The distribution of species into core or occasional categories was not significantly different from when data were ranked by persistence ($\chi_{1}^{2}$ = 0.019, *p* = 0.891). The distribution of subgroups was almost identical for occasional groups and the least abundant/persistent core group but for the abundance ranked data there were a further three core groups as opposed to one for persistence ranked.

### 3.7 *Onthophagus* (Coleoptera), Malaysia (Hanski, 1982)

**FIGURE 4:**
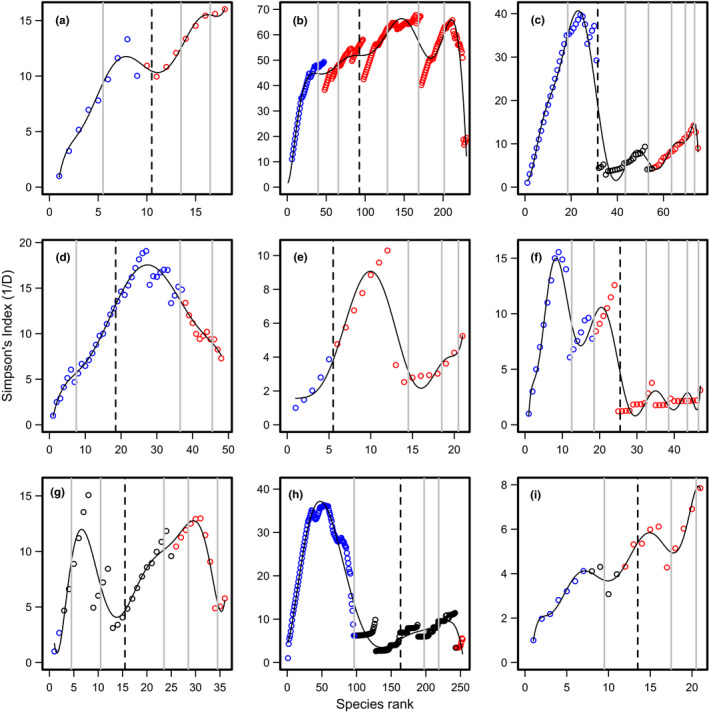
Simpson's index (1/*D*) values calculated from species abundance data, from nine previously studied datasets, ranked by persistence (except (i), which was ranked abundance) with the sequential removal of the most persistent (abundant) species. Solid lines show the best fitting polynomial models. Vertical lines show the boundaries between species groups, with the dashed line indicating the division between suggested core and occasional species groups. Species groupings identified by previous studies are shown as red for core, blue for occasional and black for intermediate species. Data from: (a) Hanski (1982); (b) Condit et al. (2012), divided into two groups by Gray et al. (2005); (c) Ulrich and Zalewski (2006); (d) Dolan et al. (2009); (e) Matthews (1985), grouped as core‐occasional by Boisnier et al. (2010); (f) Branden et al. (1994), grouped by Boisnier et al. (2010); (g) fish data from Hansen and Carey (2015); h) phytoplankton data from Hansen and Carey (2015); and (i) Supp et al. (2015)

The community was divided into eight core (in three groups) and 10 occasional species (in two groups) of *Onthophagus*, which was not significantly different ($\chi_{1}^{2}$ = 0.111, *p* = 0.739) from the nine core and nine satellite species identified by Hanski (1982) (Table 2, Figure 4a).

**TABLE 2:** Species groupings of nine additional datasets deconstructed using the sequential Simpson’s index method

Original study	Species grouping from original study	Species grouping of present study								
		C1	C2	C3	C4	C5	C6	O1	O2	O3
Hanski (1982)	Core = 9 Sat. = 9	2	3	3	—	—	—	5	5	—
Gray et al. (2005)^c^	Com. = 183 Rare = 42	29	33	40	36	—	—	27	26	39
Ulrich and Zalewski (2006)	Core = 20 Int. = 24 Sat. = 31	2	4	6	10	10	12	13	18	—
Dolan et al. (2009)	Core = 11 Occ. = 49	3	9	18	—	—	—	11	7	—
Boisnier et al. (2010)^a^ (USA)	Core = 16 Trans. = 5	1	2	4	9	—	—	5	—	—
Boisnier et al. (2010)^b^ (Australia)	Core = 29 Trans. = 18	1	3	5	6	7	—	7	6	12
Hansen and Carey (2015) (fish spp.)	Core = 11 Int. = 23 Occ. = 2	2	6	5	8	—	—	5	6	4
Hansen and Carey (2015) (phytoplankton spp.)	Core = 10 Int. = 146 Occ. = 97	35	21	34	—	—	—	67	96	—
Supp et al. (2015)	Core = 10 Int. = 4 Trans. = 7	1	3	4	—	—	—	4	9	—

Note

C = core species, O = occasional, Com. = common, Int. = intermediate; Occ. = occasional; Sat. = satellite; Trans. = transient.

^a^ Using data from Matthews (1985).

^b^ Using data from Branden et al. (1994).

^c^ Using data from BCI Condit et al. (2012).

### 3.8 Tree species, Barro Colorado Island (BCI), Panama (Gray et al., 2005)

The 225 species were divided into four core groups, totaling 138 species, and three occasional groups with 92 species (Table 2). If the most occasional group was considered equivalent to the rare category of Gray et al. (2005), which is logical given the rapid rise in Simpson's index value for species rank 1–39 shown in the polynomial model (Figure 4b), and the rest were classified as common, there was no significant difference between the two approaches ($\chi_{1}^{2}$ = 0.227, *p* = 0.633).

### 3.9 Carabidae (Coleoptera), Poland (Ulrich & Zalewski, 2006)

There were 44⋅ carabid species grouped into six core groups and 31 species in the two occasional groups (Table 2, Figure 4c). Ulrich & Zalewski (2006) suggested 20 core and 31 satellite species, with 24 considered intermediate. If the core groups were considered equivalent to the categories adopted by these authors as core and intermediate species (total 44), then the groupings are identical in size between the two approaches.

### 3.10 Tintinnid ciliates, Mediterranean Sea (Dolan et al., 2009)

The community was divided into three core groups, totaling 30 species and two occasional groups, which together had 18 species (Table 2, Figure 4d). Dolan et al. (2009) suggested 11 core and 49 occasional species (the disparity in overall totals being due to undetected data points from figure 3 of Dolan et al. (2009), the source of the data). There was a significant difference in the groupings using the two techniques ($\chi_{1}^{2}$ = 22.088, *p* < 0.001). However, the division between Dolan's core and occasional aligns almost exactly with the boundary between groups C1 and C2. Dolan et al. (2009) considered core species to be only those that were found on all 18 days sampled, whereas with my approach species found on 7 or more days were included.

### 3.11 Artificial reef fish, California (Boisnier et al., 2010)

The Californian fish community was divided into 16 core species, in four groups, and five occasional species in a single group (Table 2, Figure 3e). This was exactly the same core–occasional classification as used by Boisnier et al. (2010).

### 3.12 Artificial reef fish, Australia (Boisnier et al., 2010)

There was a total of 22 species of Australian fish in five core groups, and 25 species divided among the three occasional groups (Table 2, Figure 3f). This was not significantly different from the 29 core and 18 transient species suggested by Boisnier et al. (2010) ($\chi_{1}^{2}$ = 2.100, *p* = 0.147) (Figure 4f).

### 3.13 Lake fish, Wisconsin, USA (Hansen & Carey, 2015)

Of the 36 species of fish, 22 were core, arranged in four groups, with 15 occasional species in three groups (Table 2, Figure 4g). This was significantly different from 11 core and 25 intermediate and occasional as classified by Hansen and Carey (2015) ($\chi_{1}^{2}$ = 5.625, *p* = 0.018) (Table 2, Figure 4g). However, C1–C3 total 13 species (Table 2), which is very close to Hansen & Carey's core classification.

### 3.14 Lake phytoplankton, Wisconsin, USA (Hansen & Carey, 2015)

The phytoplankton community was divided into 90 core species, in three groups, and 156 occasional species in two groups (Table 2, Figure 4h). This was very different from the 10 core species suggested by Hansen and Carey (2015). However, the least frequent occasional grouping was of 96 species, which was almost identical to the 97 considered occasional by the source study.

### 3.15 Small mammals, Arizona, USA (Supp et al., 2015)

There were three core subgroups containing a total of eight species and two occasional groups, with 13 species (Table 2, Figure 4i). If the occasional groups were considered to be equivalent to the intermediate and transient species of Supp et al. (2015), then the groupings from the two techniques were almost identical ($\chi_{1}^{2}$ = 0.097, *p* = 0.755).

## 4 DISCUSSION

Previous plotting of abundance against persistence (i.e., frequency of occurrence) used to deconstruct communities into core and occasional species (Genner et al., 2004; Magurran & Henderson, 2003) relied on the availability of community data over many sampling events, and without this, it has been difficult to reliably distinguish these species groups (Barlow et al., 2010). However, the strong effect of group size on the number of species classified as being core makes it difficult to know which group size is most appropriate and to have confidence in the allocation of species. Moreover, this problem does not just apply to short‐term datasets but to any sample series. For example, if with the dataset from Genner et al. (2004) persistence were measured in months, half years or pairs of years instead of on an annual basis, the result using these previous methods would be a large difference in the number of core species identified. So, does that mean that the classification of core species by Magurran and Henderson (2003) and by Genner et al. (2004) was unreliable? Although that is theoretically possible, in actuality this appeared not to be the case, as the division of the Bristol Channel fish community was found to be closely aligned to the ecology of the species and their relative abundance (Magurran & Henderson, 2003). However, the problem of group size still puts into question the applicability of grouping techniques that rely on persistence in this way.

The group size problem was solved with the new method by using persistence simply as a ranking, not as a continuous variable. The community was then deconstructed based on the differential abundance of species using the property of Simpson's index (1/*D*) as a measure of the probability that any two individuals drawn at random are the same species (Magurran, 2004). Using Simpson's index on progressively smaller sections of the community is a novel approach. Boisnier et al. (2010) had done something similar before but added all species at a given level of persistence, whereas I had removed individual species, irrespective of whether they had the same persistence as the next species or not. They had also been expecting a positive, linear regression, whereas I did not have any a priori expectation of a particular model and was interested in describing the shape of the relationship and what it indicated about species groups. However, a really important outcome is that, on the assumption, a community is adequately sampled, there is no need for many years of data to divide it into core and occasional species. A particularly useful consequence of which is that by assessing the identity of species groupings over a series of shorter time periods, changes to community structure over time, including the status of individual species, can be more easily studied (Genner et al., 2004; Henderson & Magurran, 2014; Henderson et al., 2011; Magurran, 2007; Magurran & Henderson, 2010). It could help to show whether the number or proportion of species in a group changed or whether particular species switched between subgroups or from a core group to an occasional grouping or vice versa. Such changes might be related to species invasions, climate change, or other anthropogenic factors such as changing habitat management and the differential removal of species through hunting.

The sequential Simpson's index method resulted in a very similar or identical pattern of deconstruction previously identified for the Bristol Channel (Magurran & Henderson, 2003) and English Channel fish communities (Genner et al., 2004), indicating it is a reliable way of distinguishing core species from occasional. However, more than simply dividing communities into two groups the higher order polynomials of the models, with their multiple inflection points, resulted in core and occasional species, being divided into a number of subgroups. Therefore, the new method enables both a binary division and a more complex structure that may be a better reflection of reality.

One of the main advantages of the new method is that it reduces subjectivity relative to other methods of grouping core and occasional species, through identifying an optimum model based on statistical grounds. Previous methods, such as those of Hansen and Carey (2015) and Dolan et al. (2009), have had a more subjective approach, in their cases considering core species only as those that were found on all sampling occasions. Application of the new method is much more objective and is likely to be much closer to a representation of reality.

One of the criticisms of the concept of a core (i.e., common)‐occasional (i.e., satellite or rare) dichotomy has been that it is an over simplification of reality (Ulrich & Ollik, 2004; Whittaker, 2015). The new method retains the core–occasional framework while identifying of subgroups helps to describe better the patterns of species that occur between the extremes of persistence (Ugland & Gray, 1982; Ulrich & Ollik, 2004). This approach may be particularly useful in studying multimodal species abundance distributions (Antão et al., 2017; Matthews, Borges, et al., 2014).

Do subgroups have any biological or ecological meaning or are they simply a description of probability due to relative abundance of their component species? Magurran and Henderson (2003) found that in an estuarine ecosystem the core species were those associated with muddy substrates, whereas the occasional species preferred habitats, or are normally found in deep water. This showed that there was a connection between habitat association, abundance, and persistence. So, by extension, habitat preference or other ecological characteristics can be expected to align with subgroups. Indeed, Ugland and Gray (1982) suggested that communities are composed of groups of species whose constituents have some similarity in their adaptation to a habitat. Therefore, subgroups of species may have ecological meaning in relation to habitat preference and other niche characteristics.

Unfortunately for many species, particularly invertebrates, knowledge of their ecology is limited, so it may be difficult to relate this to species groupings. However, some comment can be made about the subgroups of Hemiptera and Coleoptera identified from the grassland at Grange. The Hemiptera genera, *Javesella* and *Macrosteles*, were represented by relatively unspecialized grass‐feeding species, often associated with disturbed grasslands (Nickel, 2003). Within the subgroups, both genera showed a clear abundance ranking of species. A similar pattern was found in Coleoptera of the genus *Stenus*, which are predators of soft‐bodied arthropods such as Collembola, and in *Trifolium* feeding weevils (Curculionoidea) (Lott & Anderson, 2011; Morris, 1990, 2002). The order and scale of the differences between these species may reflect differences their respective niches (Harpole & Tilman, 2006; Southwood, 1996). Many other core Hemiptera species were generalist grass feeders, and core Coleoptera were often generalist predators or associated with cattle dung (Nickel, 2003; Skidmore, 1991). Several core species could be related to the presence of their specialized food plants, such as the aphid *Thecabius affinis*, which feeds of *Ranunculus*, and the aphid *Acyrthosiphon pisum* a specialist on *Trifolium repens* and other leguminous plants (Blackman, 2010; Heie, 1980). Consequently, these Hemiptera and Coleoptera examples suggest that the subgrouping of species may well be related to their ecological niche.

The identity of the species included in the subgroups did differ depending on whether persistence or abundance was used to rank them. However, due to the close relationship between persistence and abundance (Magurran, 2004; Magurran & Henderson, 2003), the overall size and pattern of the subgroups showed little difference between models based on abundance ranking and those with persistence ranking. Consequently, abundance data alone could be used for the identification of core and occasional species and of the subgroup structure. To do so, a community would have to be well sampled and data give a good representation of true species richness (Gotelli & Colwell, 2001; Magurran, 2004). However, on that assumption, the new technique does not require long runs of sample data over many years. Therefore, data collected over short‐term periods or which are related to very long‐lived species such as trees (Barlow et al., 2010; Condit et al., 2012; Umaña et al., 2017) can be deconstructed. Furthermore, communities could be deconstructed using data from individual years allowing investigation of how species groupings change over time in relation to issues such as climate change and human impact (Genner et al., 2004; Henderson & Magurran, 2014; Henderson et al., 2011; Magurran, 2007; Magurran & Henderson, 2010). Allocation of a species to a grouping would be mathematically independent of their status in other years. This would be particularly useful in studying changes in increasing or declining species, such as the fish *Liparis liparis* in the Bristol Channel, which may have declined due to increasing water temperature (Henderson et al., 2011). The new approach could also be extended to spatial studies to identify core and satellite species and species subgroupings, assessed at the individual site scale (Matthews, Borges, et al., 2014; Supp et al., 2015).

The application of the new sequential Simpson's method to nine further datasets investigated its wider applicability. The division into core and occasional was identical or very nearly identical to the classification used by Hanski (1982), Boisnier et al. (2010) (Matthews 1985 data), Ulrich & Zalewski (2006) and Supp et al. (2015). For that of Gray et al. (2005), Dolan et al. (2009), and Boisnier et al. (2010) (Brandon 1994 data), binary core–occasional division did differ but there were strong similarities in other aspects of the groups identified. The least similarity was shown with the two Hansen and Carey (2015) datasets, who had not made a binary division but instead had grouped species at the extremes of abundance and/or persistence as core and occasional, with those species between being considered intermediate. Therefore, the new method can be successfully applied to a range of taxa, locations, and study approaches.

The division of communities into multiple groupings is aligned with the view that a binary division into core and occasional is simplistic and in reality communities are much more likely to be multimodal in structure (Antão et al., 2017; Matthews, Borges, et al., 2014). Although the new method can be used for a simple core–occasional dichotomy, it also identifies a more complex structure of multimodality. By applying the multiple groups to the previously studied datasets, rather than focusing on a simple dichotomy, reveals that there is strong agreement between the findings of the new approach and the various techniques used by other studies, even when the initial core–occasional grouping did not fit as well.

## 5 CONCLUSION

In applying the core–occasional species concept to a short‐term data set of grassland insects, I found a probability based way of dividing communities into species groups. I have demonstrated that by sequentially removing species and calculating Simpson's index reveals patterns that distinguish the same core and occasional species classification as proposed by earlier studies and so show that it can be applied to a wide range of datasets, whether based on short‐ or long‐term data. However, it has the advantage over previous methods in having no a priori assumption of a binary division of species but rather can identify multimodality in species abundance distributions. It agrees well with previous studies that have used a range of taxa and sampling methodologies and offers a more objective approach to the study of species groupings. Moreover, it does not rely on long time series of sample data and can be used even if only species abundances are known, assuming well‐sampled communities. It can therefore be applied to a far greater range of community data allowing a more fine‐scaled approach and so has the potential to provide a valuable insight into how communities change in time or space.

## CONFLICT OF INTEREST

The author has no competing interests to declare.

## AUTHOR CONTRIBUTION

**Alvin J. Helden:** Conceptualization (lead); Data curation (lead); Formal analysis (lead); Investigation (lead); Methodology (lead); Project administration (lead); Resources (lead); Software (lead); Validation (lead); Visualization (lead); Writing‐original draft (lead); Writing‐review & editing (lead).

## Data Availability

Data from Teagasc Grange can be accessed at https://doi.org/10.5061/dryad.ksn02v74g. Data from the Bristol Channel fish study are available from the Pisces Conservation website at: http://www.pisces‐conservation.com/phmm‐data‐old.html. The Bristol Channel and English Channel data are available online in an appendix to Genner et al. (2004). Permission was also given by Peter Henderson and Anne Magurran to use their fish data. Sources of data used from other published studies are described in the Section 2.

## APPENDIX A

### Appendix A1: R code used to apply the sequential Simpson's index method, followed by method used to identify model inflection points

Part 1: R code

# The following are data of species abundances of fish from the Bristol Channel. The data come from Magurran and Henderson (2003) and are ordered by the number of years found in the community (persistence)*. The data are placed in vector a. [If there is no persistence/frequency ranking known, or if only relative abundance is of interest, the values need to be ranked i.e. a<‐sort(a)]

**Table:**

a<‐c(1,1,1,1,1,1,1,1,1,1,2,2,2,3,2,2,2,2,5,3,3,5,6,4,4,4,5,24,57,75,5,9,15,7,15,18,7,7,19,22,40,17,19,11,36,88,17,31,25,34,40,109,198,49,61,172,53,88,124,60,64,220,444,601,127,238,865,943,303,366,1012,1342,2189,2236,2642,3544,3926,5122,6815,32692,37379)

# One is added to each value in vector a. This is to avoid multiplying by zero when all remaining species in a sub‐set of the community only contains singletons.

**Table:**

a=a+1

# The following code creates a series of sequentially smaller vectors, with the most abundant species being removed at each step. So, the first vector contains all species, the second contains all species except the most abundant, the third all species except the two most abundant etc. The final vector created has only the final two species in the original dataset (in most cases these would be singletons). The vectors are placed a matrix called listOutput.

**Table:**

b=length(a)‐1 listOutput<‐vector("list", length(b)) for (i in 0:b) { if (mean(a[1:(length(a)‐i)])) { output<‐a[1:(length(a)‐i)] listOutput[[i+1]]<‐output } }

# This code firstly creates a function to calculate Simpson's index (1/*D*). It then calculates the Simpson's index value for each species vector and places the output into a new vector called simp.vect2. Lastly it creates a vector called spp, which is simply a list of integers from 1 to the number of values of Simpson's index calculated.

**Table:**

SIndex<‐function(x) { 1/(sum((x*(x‐1))/((sum(x))*(sum(x)‐1)))) } simp.vect2<‐sapply(listOutput,SIndex) spp<‐c(length(a):1)

# The Simpson's index values (simp.vect2) can then be modelled with spp as the explanatory variable, for example as follows.

**Table:**

model9x<‐glm(simp.vect2~spp+I(spp^2)+I(spp^3)+I(spp^4)+I(spp^5)+I(spp^6)++I(spp^7)+I(spp^8)+I(spp^9),family=gaussian) summary(model9x)

# The data can be plotted with the following code to show a scatter plot with the linear model added.

**Table:**

plot(spp,simp.vect2,las=1,xlab="Species persistence rank",ylab="Simpson's index (1/D)",pch=16) points(spp,(((coef(model9x)["I(spp^9)"])*(spp^9))+((coef(model9x)["I(spp^8)"])*(spp^8))+((coef(model9x)["I(spp^7)"])*(spp^7))+((coef(model9x)["I(spp^6)"])*(spp^6))+((coef(model9x)["I(spp^5)"])*(spp^5))+((coef(model9x)["I(spp^4)"])*(spp^4))+((coef(model9x)["I(spp^3)"])*(spp^3))+((coef(model9x)["I(spp^2)"])*(spp^2))+((coef(model9x)["spp"])*spp)+(coef(model9x)["(Intercept)"])),type="l",las=1)

# To enable identification of inflection points in the model, the equation for the model can be solved for y for each species rank (x)

**Table:**

y<‐(((coef(model9x)["I(spp^9)"])*(spp^9))+((coef(model9x)["I(spp^8)"])*(spp^8))+((coef(model9x)["I(spp^7)"])*(spp^7))+((coef(model9x)["I(spp^6)"])*(spp^6))+((coef(model9x)["I(spp^5)"])*(spp^5))+((coef(model9x)["I(spp^4)"])*(spp^4))+((coef(model9x)["I(spp^3)"])*(spp^3))+((coef(model9x)["I(spp^2)"])*(spp^2))+((coef(model9x)["spp"])*spp)+(coef(model9x)["(Intercept)"])))

[*The values for persistence (number of years recorded) by which the abundance data in vector a were ordered are: 1,1,1,1,1,1,1,1,1,1,1,1,1,1,2,2,2,2,2,3,3,3,3,4,4,4,4,4,4,4,5,5,5,6,6,6,7,7,7,7,7,8,8,9,9,9,10,10,13,13,14,15,16,17,17,18,19,19,19,20,20,20,20,20,21,21,21,21,22,22,22,22,22,22,22,22,22,22,22,22,22]

Part 2: Identification of inflection points

With higher order polynomials, the inflection point cannot be determined by differentiation, therefore the model data need to be investigated as follows.

- The values of the model (*y*) for each species rank (*x*) are listed (see table, below – data from Supp et al. (2015)).
- The difference is taken between each value of y and the value of the preceding value i.e. *y_x_* − *y_x_*_−1_.
- The difference values change sequentially in either a positive or negative direction.
- Inflection points are represented when the difference values change direction. In the table below, the superscript letters (a‐g) indicate this, with inflection points occurring between values with different letters. For example, the difference figure decreases from species 2 to species 3, then after species 3 it starts to increase (until species 5), so an inflection point (change from a decreasing difference to increasing difference) lies somewhere between species 3 and 4.

**Table:**

Species rank (*x*)	Value of model (*y*)	Difference between *y_x_* and *y_x_*_−1_
1	0.979	
2	2.040	1.061^a^ (i.e., 2.040–0.979)
3	2.184	0.144^a^
4	2.610	0.426^b^
5	3.292	0.683^b^
6	3.876	0.584^c^
7	4.118	0.242^c^
8	4.022	−0.096^c^
9	3.787	−0.235^c^
10	3.675	−0.112^d^
11	3.871	0.196^d^
12	4.393	0.522^d^
13	5.076	0.683^d^
14	5.643	0.567^e^
15	5.838	0.195^e^
16	5.588	−0.250^e^
17	5.120	−0.468^e^
18	4.953	−0.167^f^
19	5.649	0.696^f^
20	7.184	1.536^f^
21	7.790	0.605^g^

### Appendix A2: Details of final models for the 13 datasets used in the study

**Bristol Channel fish**

When ranked by persistence a 14th order polynomial showed the best fit. The glm model was as follows:

*y* = 3.593e^−1^ + 3.345e^−1^ *x* ^3^ − 9.269 e^−2^ *x* ^4^ + 1.160e^−2^ *x* ^5^ − 8.424e^−4^ *x* ^6^ + 3.912e^−5^ *x* ^7^ − 1.220e^−6^ *x* ^8^ + 2.620e^−8^ *x* ^9^ − 3.883e^−10^ *x* ^10^ + 3.909e^−12^ *x* ^11^ − 2.553e^−14^ *x* ^12^ + 9.764e^−17^ *x* ^13^ − 1.661e^−19^ *x* ^14^

In this model, there were 10 inflection points, but the three least persistent were combined giving a division of the community into nine groups. Four groups were considered more persistent, core groups, together containing 99.4% of all individuals, and there were five less persistent, occasional groups.

When ranked by abundance, data were best described by a ninth order polynomial. The glm model was as follows:

*y* = −2.111 + 2.531*x* − 1.313e^−2^ *x* ^3^ + 1.239e^−3^ *x* ^4^ − 5.846e^−5^ *x* ^5^ + 1.523e^−6^ *x* ^6^ − 2.206e^−8^ *x* ^7^ + 1.663e^−10^ *x* ^8^ − 5.081e^−13^ *x* ^9^

Deconstructing the community resulted in three core groups (C1 = 5, C2 = 9, C3 = 16) totaling 30 species and 99.4% of individuals, and two occasional groups (O1 = 16, O2 = 35) totaling 51 species.

**English Channel fish**

The best fitting model for persistence ranked data was a tenth order polynomial. The community was split into four core groups, containing 99.7% of all individuals, and four occasional groups. The glm model was as follows:

*y* = 6.488 − 7.674*x* + 3.746*x* ^2^ − 0.628e^−1^ *x* ^3^ + 5.263e^−2^ *x* ^4^ − 2.552e^−3^ *x* ^5^ + 7.564e^−5^ *x* ^6^ − 1.391e^−6^ *x* ^7^ + 1.549e^−8^ *x* ^8^ − 9.556e^−11^ *x* ^9^ + 2.508e^−13^ *x* ^10^

The abundance rank data were best described with a tenth order polynomial model. The glm model was as follows:

*y* = 2.413 − 2.620*x* + 1.912*x* ^2^ − 3.391e^−1^ *x* ^3^ + 2.949e^−2^ *x* ^4^ − 1.470e^−3^ *x* ^5^ + 4.474e^−5^ *x* ^6^ − 8.451e^−7^ *x* ^7^ + 9.671e^−9^ *x* ^8^ − 6.139e^−11^ *x* ^9^ + 1.659e^−13^ *x* ^10^

The deconstructed community grouping was for five core groups (C1 = 1, C2 = 8, C3 = 10, C4 = 10, C5 = 12), totaling 41 species and 99.9% of individuals and four occasional groups (O1 = 8, O2 = 11, O3 = 6, O4 = 6), with 31 species.

**Grange Hemiptera**

The model that best fitted the persistence ranked data was a 16th order polynomial. The glm model was as follows:

*y* = −3.125e^−1^ + 2.238*x* ^3^ − 1.736*x* ^4^ + 6.260e^−1^ *x* ^5^ − 1.336e^−1^ *x* ^6^ + 1.861e^−2^ *x* ^7^ − 1.787e^−3^ *x* ^8^ + 1.218e^−4^ *x* ^9^ −5.989e^−6^ *x* ^10^ + 2.131e^−7^ *x* ^11^ − 5.436e^−9^ *x* ^12^ + 9.688e^−11^ *x* ^13^ − 1.145e^−12^ *x* ^14^ + 8.066e^−15^ *x* ^15^ − 2.562e^−17^ *x* ^16^

The community was split into six core groups containing 98.5% of individuals and three occasional groups.

The abundance ranked data were best described with a tenth order polynomial. The glm model was as follows:

*y* = 1.336 + 2.714e^−1^ *x* ^3^ + 7.350e^−2^ *x* ^4^ + 8.777e^−3^ *x* ^5^ − 5.773e^−4^ *x* ^6^ + 2.230e^−5^ *x* ^7^ − 5.043e^−7^ *x* ^8^ + 6.186e^−9^ *x* ^9^ − 3.181e^−11^ *x* ^10^

The community was deconstructed into four core groups (C1 = 2, C2 = 5, C3 = 4, C4 = 4) totaling 15 species and representing 98.2% of the individuals, and three occasional groups (O1 = 10, O2 = 10, O3 = 6), with 26 species overall.

**Grange Coleoptera**

The best fitting model for persistence ranked data was an 11th order polynomial. The glm model was as follows:

*y* = 7.362e^−1^ + 6.010e^−1^ *x* ^2^ − 7.751e^−2^ *x* ^3^ + 5.274e^−3^ *x* ^4^ − 2.091e^−4^ *x* ^5^ + 5.097e^−6^ *x* ^6^ − 7.866e^−8^ *x* ^7^ + 7.711e^−10^ *x* ^8^ − 4.655e^−12^ *x* ^9^ + 1.579e^−14^ *x* ^10^ − 2.306e^−17^ *x* ^11^

There were seven inflection points, and the community was divided into five species groups: two core groups and three occasional. The two core groups contained 94.7% of individuals.

A 12th order model best described the pattern in the abundance ranked data. The glm model was as follows:

*y* = −1.550 + 2.142*x* − 2.508e^−4^ *x* ^4^ + 3.125e^−5^ *x* ^5^ − 1.662e^−6^ *x* ^6^ + 4.796e^−8^ *x* ^7^ − 8.237e^−10^ *x* ^8^ + 8.684e^−12^ *x* ^9^ − 5.523e^−14^ *x* ^10^ + 1.948e^−16^ *x* ^11^ − 2.931e^−19^ *x* ^12^

There were four core groups (C1 = 9, C2 = 3, C3 = 11 and C4 = 16) totaling 39 species and including 95.0% of all individuals, and three occasional groups (O1 = 21, O2 = 21, O3 = 44).

***Onthophagus* (Coleoptera), Malaysia (** **Hanski,** **1982** **)**

*y* = −7.846 + 14.480*x* − 7.533*x* ^2^ + 2.098*x* ^3^ − 3.031e^−1^ *x* ^4^ + 2.308e^−2^ *x* ^5^ − 8.799e^−4^ *x* ^6^ + 1.325^−5^ *x* ^7^

**Tree species, Barro Colorado Island (BCI), Panama (** **Gray et al.,** **2005** **)**

*y* = 1.390 + 3.362e^−1^ *x* ^2^ − 2.110e^−2^ *x* ^3^ + 6.029e^−4^ *x* ^4^ − 9.743e^−6^ *x* ^5^ + 9.570e^−8^ *x* ^6^ − 5.816e^−10^ *x* ^7^ + 2.134e^−12^ *x* ^8^ − 4.323e^−15^ *x* ^9^ + 3.710e^−18^ *x* ^10^

**Carabidae (Coleoptera), Poland (** **Ulrich & Zalewski,** **2006** **)**

*y* = −1.328 + 2.060e^−1^ *x* ^3^ − 4.792^−2^ *x* ^4^ + 5.040e^−3^ *x* ^5^ − 3.007^−4^ *x* ^6^ + 1.107^−5^ *x* ^7^ − 2.608^−7^ *x* ^8^ + 3.942^−9^ *x* ^9^ − 3.703^−14^ *x* ^10^ + 1.970e^−13^ *x* ^11^ − 4.535^−16^ *x* ^12^

**Tinitinnid ciliates, Mediterranean Sea (** **Dolan et al.,** **2009** **)**

*y* = −1.058 + 2.340*x* − 3.631e^−1^ *x* ^2^ + 3.046 e^−2^ *x* ^3^ − 1.157e^−3^ *x* ^4^ + 1.982e^−5^ *x* ^5^ − 1.262e^−7^ *x* ^6^

**Artificial reef fish, California (** **Boisnier et al.,** **2010** **)**

*y* = 1.569 + 1.900e^−3^ *x* ^5^ − 4.183e^−4^ *x* ^6^ + 3.439e^−5^ *x* ^7^ − 1.251e^−6^ *x* ^8^ + 1.700e^−8^ *x* ^9^

**Artificial reef fish, Australia (** **Boisnier et al.,** **2010** **)**

*y* = −10.690 + 20.220*x* − 11.650*x* ^2^ + 3.492*x* ^3^ − 5.659e^−1^ *x* ^4^ + 5.405e^−2^ *x* ^5^ − 3.225e^−3^ *x* ^6^ + 1.237e^−4^ *x* ^7^ − 3.051e^−6^ *x* ^8^ + 4.678e^−8^ *x* ^9^ − 4.060e^−10^ *x* ^10^ + 1.524e^−12^ *x* ^11^

**Lake fish, Wisconsin, USA (** **Hansen & Carey,** **2015** **)**

*y* = 9.970 − 13.930*x* + 7.086*x* ^2^ − 1.356*x* ^3^ + 1.298e^−1^ *x* ^4^ − 6.905e^−3^ *x* ^5^ + 2.078e^−4^ *x* ^6^ − 3.315e^−6^ *x* ^7^ + 2.180e^−8^ *x* ^8^

**Lake phytoplankton, Wisconsin, USA (** **Hansen & Carey,** **2015** **)**

*y* = 4.063 + 4.585e^−1^ *x* + 4.782e^−2^ *x* ^2^ − 1.555e^−3^ *x* ^3^ + 1.791e^−5^ *x* ^4^ − 9.939e^−8^ *x* ^5^ + 2.701e^−10^ *x* ^6^ − 2.893e^−13^ *x* ^7^

**Small mammals, Arizona, USA (** **Supp et al.,** **2015** **)**

*y* = −4.953 + 10.810*x* − 6.615*x* ^2^ + 2.051*x* ^3^ − 3.449e^−1^ *x* ^4^ + 3.290e^−2^ *x* ^5^ − 1.776e^−3^ *x* ^6^ + 5.049^−5^ *x* ^7^ − 5.866e^−7^ *x* ^8^

### Appendix A3: Persistence range, measured as the number of times species were present in samples (maximum value shown in column title), also expressed as a percentage (in brackets), and abundance range shown by each of the species groups identified by deconstructing the Hemiptera and Coleoptera communities from Grange

**Table:**

Species grouping	Hemiptera		Coleoptera	
	Persistence/115 (%)	Abundance	Persistence/63 (%)	Abundance
C1	115 (100)	2,672	29–63 (46.0–100)	43–1,957
C2	102 (88.7)	551	15–28 (23.8–44.4)	16–33
C3	71–80 (61.7–69.6)	168–224	—	—
C4	54–66 (47.0–57.4)	117–345	—	—
C5	21–53 (18.3–46.1)	26–456	—	—
C6	11–20 (9.6–17.4)	24–28	—	—
O1	3–10 (2.6–8.7)	7–11	5–14 (7.9–22.2)	5–17
O2	2–3 (1.7–2.6)	2–7	3–4 (4.7–6.3)	3–6
O3	1 (0.9)	1–2	1–2 (1.6–3.2)	1–3

### Appendix A4: List of species of Hemiptera and Coleoptera from Grange, contained within each group identified by deconstructing the communities. Within each group, species are ranked first by persistence (frequency), starting with the most persistent at the top, and then alphabetically

**Table:**

Hemiptera species	Hemiptera group	Coleoptera species	Coleoptera group
*Rhopalosiphum* Koch sp.	C1	*Mocyta fungi* (Gravenhorst)	C1
*Metopolophium* Mordvilko sp.	C2	*Amischa analis* (Gravenhorst)	C1
*Myzus* Passerini sp.B	C3	*Aloconota gregaria* (Erichson)	C1
*Myzus* Passerini sp.A	C3	*Stenus formecitorum* Mannerheim	C1
*Javesella obscurella* (Boheman)	C4	*Stenus nanus* Stephens	C1
*Thecabius affinis* (Kaltenbach)	C4	*Sitona lepidus* (Fabricius)	C1
*Macrosteles viridigriseus* (Edwards)	C4	*Ptenidium pusillum* (Gyllenhal)	C1
*Sipha glyceriae* (Kaltenbach)	C5	*Megasternum concinnum* (Marsham)	C1
*Javesella pellucida* (Fabricius)	C5	*Tachyporus pusillus* Gravenhorst	C1
*Sitobion avenae* (Fabricius)	C5	*Stenus clavicornis* (Scopoli)	C1
*Macrosteles sexnotatus* (Fallén)	C5	*Acrotrichis atomaria* (De Geer)	C1
*Microlophium evansi* (Theobald)	C6	*Amischa decipiens* (Sharp)	C1
*Sitobion fragariae* (Walker)	C6	*Pterostichus strenuus* Panzer	C1
*Acyrthosiphon pisum* (Harris)	C6	*Atomaria nitidula* (Marsham)	C1
*Macrosteles laevis* (Ribaut)	C6	*Stenus picipes* Stephens	C1
*Uromelan* (Mordvilko) sp.	C6	*Bembidion lampros* (Herbst)	C1
*Saldula orthochila* (Fieber)	O1	*Tachyporus chrysomelinus* (L.)	C1
*Atheroides serrulatus* Haliday	O1	*Amischa nigrofusca* (Stephens)	C1
*Javesella dubia* (Kirschbaum)	O1	*Ptenidium nitidum* (Heer)	C1
*Anthocoris nemorum* (L.)	O1	*Longitarsus luridus* (Scopoli)	C1
*Aphrodes albifons* (L.)	O1	*Protapion fulvipes* (Geoffroy)	C1
*Holcaphis* Hille Ris Lambers sp.	O2	*Philonthus* *carbonarius* (Gravenhorst)	C2
Unidentified aphid sp.G	O2	*Stenus cicindeloides* (Schaller)	C2
*Philaenus spumarius* (L.)	O2	*Acrotona* Thomson, C.G. sp.A	C2
*Pithanus maerkeli* (Herrich‐Schäffer)	O2	*Stenus brunnipes* Stephens	C2
*Phyllaphis fagi* (L.)	O2	*Bembidion guttul*a (Fabricius)	C2
*Nabis ferus* (L.)	O2	*Oxypoda* Mannerheim sp.E	C2
*Myzus cerasi* (Fabricius)	O2	*Gabrius appendiculatus* Sharp	C2
*Drymus sylvaticus* (Fabricius)	O2	*Stenus similis* (Herbst)	C2
*Brachycaudus helichrysi* (Kaltenbach)	O2	*Tachyporus dispar* (Paykull)	C2
*Aphrodes makarovi* Zakhvatkin	O2	*Pterostichus vernalis* (Panzer)	C2
*Aphis* L. sp.	O2	*Ptiliolum spencei* (Allibert)	C2
*Pachytomella parallela* (Meyer‐Dür)	O3	*Tachyporus hypnorum* (Fabricius)	C2
*Streptanus sordidus* (Zetterstedt)	O3	*Acrotrichis grandicollis* (Mannerheim)	C2
*Stenodema calcarata* (Fallén)	O3	*Bembidion aeneum* Germar	C2
*Saldula saltatoria* (L.)	O3	*Philonthus* *cognatus* Stephens	C2
*Psylla melanoneura* Förster	O3	*Tachyporus nitidulus* (Fabricius)	C2
*Paraliburnia clypealis* (J. Sahlberg)	O3	*Atomaria apicalis* Erichson	C2
*Megophthalmus scabripennis* Fallén	O3	*Stenus canaliculatus* Gyllenhal	O1
*Aphalara exilis* (Weber & Mohr)	O3	*Cartodere nodifer* (Westwood)	O1
*Acyrthosiphon* Mordvilko sp.A	O3	*Stenus ossium* Stephens	O1
		*Helophorus brevipalpis* Bedel	O1
		*Atomaria atricapilla* Stephens	O1
		*Stephostethus* *lardarius* (De Geer)	O1
		*Quedius schatzmayri* Gridelli	O1
		*Ceutorhynchus erysimi* (Fabricius)	O1
		*Anotylus tetracarinatus* (Block)	O1
		*Oligota* Mannerheim sp.	O1
		*Stenus fulvicornis* Stephens	O1
		*Ischnopterapion virens* (Herbst)	O1
		*Nebria brevicollis* (Fabricius)	O1
		*Cercyon melanocephalus* L.	O1
		*Xantholinus linearis* (Olivier)	O1
		*Gabrius breviventer* (Sperk)	O1
		*Enicmus histrio* Joy & Tomlin	O1
		*Xantholinus longiventris* Heer	O1
		*Tachinus laticollis* Gravenhorst	O1
		*Philonthus* *laminatus* (Creutzer)	O1
		*Cercyon impressus* (Sturm)	O1
		*Agonum muelleri* (Herbst)	O2
		*Trechus quadristriatus* (Schrank)	O2
		*Tachyporus obtusus* (L.)	O2
		*Rhinoncus pericarpius* (L.)	O2
		*Pteryx suturalis* (Heer)	O2
		*Philonthus* *marginatus* (Müller)	O2
		*Loricera pilicornis* (Fabricius)	O2
		*Dimetrota* Mulsant & Rey sp.B	O2
		*Dimetrota* Mulsant & Rey sp.A	O2
		*Datomicra* Mulsant & Rey sp.A	O2
		*Clivina fossor* (L.)	O2
		*Cercyon pygmaeus* (Illiger)	O2
		*Tachinus rufipes* (L.)	O2
		*Leiosoma deflexum* (Panzer)	O2
		*Coccidula rufa* (Herbst)	O2
		*Stenus juno* (Paykull)	O2
		*Philhygra* Mulsant & Rey sp.C	O2
		*Microdota* Mulsant & Rey sp.B	O2
		*Cortinicara gibbosa* (Herbst)	O2
		*Ceutorhynchus typhae* (Paykull)	O2
		*Autalia rivularis* (Gravenhorst)	O2
		*Aleochara lanuginosa* Gravenhorst	O2
		*Platystethus* *arenarius* (Fourcroy)	O3
		*Malthodes pumilus* (Brébisson)	O3
		Unknown Aleocharinae sp.A	O3
		*Tachyporus solutus* Erichson	O3
		*Pterostichus melanarius* (Illiger)	O3
		*Ptenidium fuscicorne* Erichson	O3
		*Propylea quattuordecimpunctata* (L.)	O3
		*Oxytelus laqueatus* (Marsham)	O3
		*Microdota* Mulsant & Rey sp.E	O3
		*Lema cyanella* (L.)	O3
		*Encephalus complicans* Stephens	O3
		*Cypha* Leach sp.	O3
		*Cercyon haemorrhoidalis* (Fabricius)	O3
		*Anotylus rugosus* (Fabricius)	O3
		*Xantholinus punctulatus* (Paykull)	O3
		*Typhaea stercorea* (L.)	O3
		*Tychus niger* (Paykull)	O3
		*Tachyporus tersus* Erichson	O3
		*Rugilus similis* (Erichson)	O3
		*Quedius nitipennis* (Stephens)	O3
		*Philonthus* *varians* (Paykull)	O3
		*Philonthus* *intermedius* (Lacordaire)	O3
		*Philhygra* Mulsant & Rey sp.D	O3
		*Oxypoda* Mannerheim sp.C	O3
		*Oxypoda* Mannerheim sp.A	O3
		*Omonadus floralis* (L.)	O3
		*Ischnosoma splendidum* (Gravenhorst)	O3
		*Micropeplus porcatus* (Paykull)	O3
		*Micrambe vini* (Panzer)	O3
		*Meligethes aeneus* (Fabricius)	O3
		*Megarthrus depressus* (Paykull)	O3
		*Megarthrus denticollis* (Beck)	O3
		*Datomicra* Mulsant & Rey sp.D	O3
		*Chaetida longicornis* (Gravenhorst)	O3
		*Cercyon nigriceps* (Marsham)	O3
		*Carpelimus bilineatus* (Stephens)	O3
		*Cantharis nigra* (De Geer)	O3
		*Calathus* *melanocephalus* (L.)	O3
		*Brachypterus urticae* (Fabricius)	O3
		*Barypeithes pellucidus* (Boheman)	O3
		*Atomaria umbrina* (Gyllenhal)	O3
		*Atheta* Thomson, C.G. sp.B	O3
		*Amara aenea* (De Geer)	O3
		*Acrotrichis sericans* (Motschulsky)	O3
